# Modelling Thermal Conduction in Polydispersed and Sintered Nanoparticle Aggregates

**DOI:** 10.3390/nano12010025

**Published:** 2021-12-22

**Authors:** Nikolaos P. Karagiannakis, Eugene D. Skouras, Vasilis N. Burganos

**Affiliations:** 1 Institute of Chemical Engineering Sciences (ICE-HT), Foundation for Research and Technology, Hellas (FORTH), GR-26504 Patras, Greece; nick_karag@iceht.forth.gr (N.P.K.); Eugene.Skouras@iceht.forth.gr (E.D.S.); 2 Department of Mechanical Engineering, University of the Peloponnese, GR-26334 Patras, Greece

**Keywords:** nanofluid, heat conduction, effective thermal conductivity, particle aggregates, polydispersity, sintering

## Abstract

Nanoparticle aggregation has been found to be crucial for the thermal properties of nanofluids and their performance as heating or cooling agents. Most relevant studies in the literature consider particles of uniform size with point contact only. A number of forces and mechanisms are expected to lead to deviation from this ideal description. In fact, size uniformity is difficult to achieve in practice; also, overlapping of particles within aggregates may occur. In the present study, the effects of polydispersity and sintering on the effective thermal conductivity of particle aggregates are investigated. A simulation method has been developed that is capable of producing aggregates made up of polydispersed particles with tailored morphological properties. Modelling of the sintering process is implemented in a fashion that is dictated by mass conservation and the desired degree of overlapping. A noticeable decrease in the thermal conductivity is observed for elevated polydispersity levels compared to that of aggregates of monodisperse particles with the same morphological properties. Sintered nanoaggregates offer wider conduction paths through the coalescence of neighbouring particles. It was found that there exists a certain sintering degree of monomers that offers the largest improvement in heat performance.

## 1. Introduction

Numerous contemporary applications are based on the incorporation of nanoparticles into convectional fluids [1,2]. The resulting nanofluids may have drastically increased thermal properties and reduced sedimentation. Nanofluids and nanoparticles are increasingly used in a variety of fields. Heating and cooling systems using nanofluids show significant improvement in energy consumption, while new medical techniques are developing using nanoparticles in bioliquids. [3,4]. A large number of recent publications study the potential use of nanofluids in multidisciplinary fields [5,6]. Although much effort has been placed on the correlation of the heat transfer properties of nanofluids with the underlying phenomena, it appears that there is no widely accepted explanation of their behaviour or a reliable way to predict their heat conduction properties [7,8].

Many models have been developed to predict the effective conductivity of nanoparticles. The effect of Brownian motion, interfacial resistance, the existence of nanolayers, and the aggregation mechanism have been discussed in detail in the literature [9,10,11,12,13]. A large increase in effective thermal conductivity has been detected experimentally when nanoparticles are organized in small aggregates [14,15]. The increased contact of the particles within the aggregate was found to facilitate heat transfer compared to fully dispersed particles. On the contrary, larger mass aggregates have a negative effect on the stability of the nanofluid and, therefore, on heat transport properties [16]. Lotfizadeh et al. [11], Prasher et al. [17], Evans et al. [18], and Liao et al. [19], among others, developed models to predict the thermal conductivity of nanofluids based on the morphology of the aggregates. These works showed that the configuration of the nanoparticles and the morphological parameters of the aggregates can alter the effective conductivity of the nanofluids noticeably. A typical assumption made in several studies is that the nanoparticles are of the same size and the neighbouring monomers are mainly at single point contact.

In real conditions, samples always come with a certain distribution in particle size [20,21,22], which is held responsible for altering the thermal properties of nanofluids [21]. It has been noticed that polydispersity may occur during dispersion of the nanoparticles in the base fluid [23,24]. Chon et al. [21] used commercial, uniform nanoparticles for the preparation of nanofluids. They measured the size distribution after dispersion and found a significant deviation. In fact, it is technically challenging to synthesize and disperse a large quantity of highly monodispersed nanoparticles [25]. Zhiting et al. [26] studied the effect of polydispersity, among other parameters, on the heat transfer coefficient of nanocomposites with molecular dynamics simulations. They concluded that polydispersity negatively affects the effective conductivity. However, the nature of the used method does not allow the simulation of large systems, such as that of aggregated nanoparticles.

Strong electrostatic forces between particles, collision of particles during the formation of the aggregates, and high-temperature environments are some of the factors that contribute to a certain degree of overlapping between particles [27,28]. Two methods have been widely used for the preparation of nanofluids. The one-step nanoparticle production and blending method produces nanofluids with increased stability and offers elevated thermal conductivity, but has a relatively high production cost and is not yet suitable for large-scale production [29]. The two-step method is one that proceeds in two sequential steps, namely, the step of separate production of nanoparticles followed by suspension in a base fluid [29]. Commercial nanoparticles are usually found in powder form. The production of nanoparticles commences with the creation of a nanoparticle suspension from their precursors. This suspension is dried using various methods and, eventually, a powdered form of nanoparticles is obtained [23,30]. Thermal decomposition of organic precursors is a well-established process for the fabrication of solid nanoparticles [23]. It is temperature dependent and has been reported to result in sintered and/or polydispersed particles [23,24,30]. Other methods include nanoparticle production in the gas phase [31,32]. During the creation of these nanoparticles, formation processes, including surface reactions, condensation, coagulation and sintering, are some of the key mechanisms that take place [32,33,34,35]. The kinetics of each process determines the final structure morphology, which can vary among spherical particles, agglomerates, or compact aggregates [34]. The resulting system usually includes partially coalesced particles with sintering necks [32,35].

Attempts to model the aforementioned process usually start with the formation of an aggregate using a stochastic method. Eggersdorfer et al. [36] modelled the sintering process in aggregates, formed by Diffusion Limited Aggregation (DLA), Diffusion Limited Cluster–Cluster Aggregation (DLCCA), and Ballistic Aggregation (BA). The driving force for sintering was the minimization of free energy. They noted that, during sintering, primary particles approach each other. Sander et al. [31] presented an analytical description of the underlying phenomena during the production of nanoparticles, such as coagulation and sintering. The primary particles were modelled having a spherical shape and a polydispersed size, with each particle described by its sintering level and radius. The final structure has been compared with experimental data and transmission electron microscopy (TEM) images. An overlapping algorithm, developed by Brasil et al. [27], has studied the effect of sintering on the morphological properties of the aggregates, such as the fractal dimension and the radius of gyration. Schmid et al. [33,37] have developed a model for aggregates subjected to coagulation and sintering. The sintered aggregates were presented as the result of successive overlapping of spherical, primary particles.

The previous discussion underlines two major open issues in the study of the thermal conductivity of aggregated nanoparticles. Even though the effect of aggregation has been extensively studied, there is a dearth of research dealing with the effect of polydispersity of the nanoparticles within the aggregate. Moreover, sintering and the concomitant partial coalescence are most likely to occur in nanoparticle systems, yet the study of their effects on heat conduction remains a challenging, open field. These configurations complicate the determination of heat transfer properties and, therefore, reliable simulations of transport phenomena are required.

The present work examines the effects of polydispersity and sintering of particles on the effective thermal conductivity of nanofluids that contain particle aggregates. To this end, the method developed by the authors [38] for reconstructing particle aggregates is extended to include particle overlapping due to sintering as well as non-uniform particle size as a realistic outcome of nanofluid preparation. Among the merits of this method is the algorithmically rapid reconstruction of agglomerated systems with predetermined properties, namely, the fractal dimension and the average number of particles in the aggregates. As a case study, the particle size here follows the normal distribution and the standard deviation is expressed as a fraction of the mean size. Moreover, a technique has been developed to simulate sintered aggregates. The sintering process is expected to change the particle position and size while, naturally, preserving the mass of the working sample. An overlap parameter and the morphology of the primary aggregate determine the final morphology. The effective thermal conductivity is calculated through the temperature distribution obtained from the solution of the heat transfer equation. The Meshless Local Petrov–Galerkin (MLPG) method [39,40,41] is used here as it was shown to provide stable and fast solutions to particulate systems even with point contact. The Discretisation-Corrected Particle Strength Exchange (DC PSE) method [42,43] is used to approach the field function and its derivatives, while the meshless nature of the method allows local increase of the domain discretisation at the interface between the base fluid and solid particles.

The effect of the overlap parameter and the polydispersity level of particle aggregates on the thermal conductivity is studied by changing the number of particles in the aggregate, the fractal dimension of the aggregates, and the volume fraction of the particles. The effective conductivity of the polydispersed nanoaggregates, as predicted by the present method, is compared to the effective conductivity of the corresponding systems, as these result from the Diffusion Limited Aggregation (DLA) method. Moreover, the effective conductivity of aggregates consisting of polydispersed particles is compared with that of aggregates of monodispersed particles, keeping all other morphological parameters constant. Notable deviations between monodispersed and polydispersed cases are observed and discussed. In addition, the effect of sintering is examined by varying the overlap parameter. The results are compared with predictions of analytic expressions from the literature.

## 2. Modelling Polydispersed and Sintered Aggregates

Modelling of fractal aggregates is the first stage in the process of correlating numerically their physical properties with their morphological parameters. In reality, aggregation is a very complex process, being sensitive to parameters such as the temperature, the physical properties of the particles and the solvent, the polydispersity extent, the primary particle shapes, etc. [11,12]. A well-established approach to generate fractal structures numerically is the usage of various stochastic methods, such as Diffusion Limited Aggregation (DLA), Diffusion Limited Cluster–Cluster Aggregation (DLCCA), and Reaction Limited Aggregation (RLA) [43,44]. The validity of these methods has been verified through comparison of the resulting structures with experimental data [45,46]. Different physical processes are engaged in different aggregation models. A very common description of such clustering relies on the fractal dimension, $d_{f}$, using the relation between the number of particles in the aggregate, N, and basic cluster-size characteristics [45,46]: *N*.

(1) $$N=k_{g}{\left(R_{g}/r_{p}\right)}^{d_{f}},$$

where $r_{p}$ is the mean radius of the primary particles, $k_{g}$ is the structure factor, and $R_{g}$ is the radius of gyration of the aggregate [12,32,47]:

(2) $$R_{g}=\sqrt{\frac{\sum_{i}^{N}m_{i}{\left(r_{i}-r_{c}\right)}^{2}}{\sum_{i}m_{i}}},$$

where $r_{i}\;$ is the position vector of the centre of mass of particle i, $m_{i}$ is its mass, and $r_{c}$ is the position vector of the centre of mass of the aggregate.

In order to study the effect of polydispersity on thermal conductivity, the work developed in [38] has been extended to include polydispersed particles. This technique offered fast convergence of the algorithm for the representation of agglomerated systems with predetermined properties. For the sake of completeness, the major steps of the algorithm are mentioned below. The primary input of the algorithm consists of the volume fraction of the particles, the fractal dimension or a range of values around it, and the average number of particles per aggregate or a range of values around it. A random deposition of a particle initiates the process. A new particle stochastically appears on the surface of the particle, and the process is repeated, with the restriction of no overlapping between any pair of particles. During the process, certain restrictions are imposed as described in [38], aiming at the convergence of the fractal dimension to the target value or range of values. It has been shown that the desired fractal dimension can be achieved with only a few particles. The process ends when the aggregate acquires a predefined number of particles. Then, another particle appears at a random place in the computational domain, and the aforementioned process is repeated for the formation of a second cluster. The whole algorithm is repeated as many times as needed to satisfy the desired number of aggregates in the working domain. Further analytical descriptions of the method are presented in [38].

In the present study, all simulations take place in a cubic box of length l. All spatial parameters and variables are normalized with this quantity. Thus, the particle radius is related with the volume fraction ($f_{p}$), the number of particles in the aggregate (N), and the number of aggregates ($N_{c}$), as follows:

(3) $$r_{p}={\sqrt[{3}]{\frac{3f_{p}}{4\pi \sum_{i}^{N_{c}}N_{i}}}}^{\;},$$

**Figure 1:**
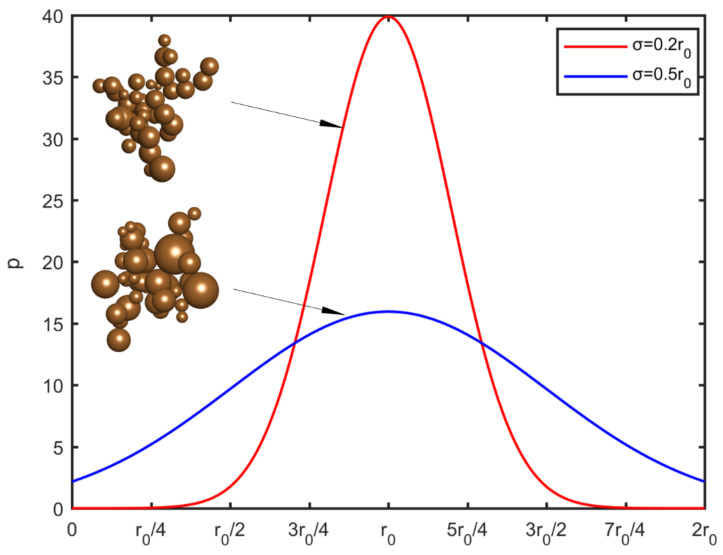
The probability density function, *p*, of the particle radius distribution for different standard deviation values (red $\sigma =0.2r_{0}$, blue $\sigma =0.5r_{0}$ ) and a representative visualization of the resulting aggregates.

For monodispersed particles, the definition of $r_{p}$ is straightforward. For polydispersed particles, in the present extended approach, this value is set as the mean radius of the particles, $r_{0}$. The exact radius of each particle is randomly sampled from a prescribed normal distribution. The deviation of the particle size distribution is expressed as a fraction of the particle radius. The probability density function $\left(p\right)$ for the particle radius is shown in Figure 1 for two different standard deviations. In order to avoid negative values, a threshold was imposed at zero radius. For symmetry reasons, another threshold is set at the value $r_{p,max}=2r_{0}$. The maximum deviation of the particle radius, in this study, is $\sigma =0.5r_{0}$. For higher polydispersity levels a lognormal distribution can be used instead, in order to avoid a large number of negative values and adhere to more realistic particle size distributions.

The resulting system may have a volume fraction different from the desired one. This is an important issue in polydispersed particle systems since large sizes may be sampled as the tail additions to a cluster. Depending on whether the desired volume fraction is smaller or greater than expected, particles will be added or removed. To remove a particle, an aggregate is randomly selected and the last-added particle is removed. To add a particle, an aggregate is randomly selected and a new particle with a size that is sampled from the prescribed distribution is added at a random location on the surface of a randomly selected particle of the aggregate, with restrictions in order to satisfy the fractal dimension and the non-overlapping condition, as described in [38] for uniformly sized particles. The process is repeated until the predetermined volume fraction is achieved. With this methodology, critical quantities such as the volume fraction, the mean radius of the particles, and the fractal dimension remain within their prescribed bounds while a small deviation is maintained in the number of particles per aggregate.

The structure factor, $k_{g}$, is strongly dependent on the polydispersity levels and the degree of overlapping of the particles [48,49]. Eggersdorfer et al. [49] and Tomchuk et al. [48] studied the effect of polydispersity on the fractal dimension and the structure factor, considering a wide range in the number of particles in each aggregate, the prescribed deviation of the particle size, and the aggregation model that is used. They noted a reduction in the structure factor for increased polydispersity for aggregates formed by the DLCCA algorithm and the Ballistic Aggregation model. Independently of the agglomeration mechanism, in the limiting case of infinitely polydispersed particles, the structure factor tends to unity [49]. For monodispersed particles with fractal dimension ranging between 1.7 and 2.5, the structure factor can be considered constant, $k_{g}=1.5$ [38,50]. In the present case, for polydispersed particles, the structure factor changes linearly with the deviation of the particle radius, taking values between 1.2 and 1.5 [49].

During sintering, particles are expected to increase their radius and come closer to each other [37]. Typical simulations of coagulation and sintering of nanoaggregates include an overlapping step, where neighbouring particles penetrate each other, and a growth step, during which particle size increases to maintain mass and volume. This process captures the redistribution of mass in the free surface of the aggregate and offers a realistic representation of the final morphology [33]. Assuming aggregates consisting of monodispersed particles and following this methodology, an overlapping coefficient is defined as [27]:

(4) $$\delta =1-{\frac{d}{2R_{p}}}^{\;},$$

where d is the final distance of the centres of the neighbour particles, and $R_{p}$ is the final radius of the particles. The initial radius of the particles can be calculated from Equation (3).

At initial stages of the sintering algorithm, aggregates are forced to collapse to their centre of mass by the penetration coefficient, $\delta_{p}$, while the sizes of the particles remain constant. The penetration coefficient relates the final distance of the neighbouring particles to the initial radius ($\delta_{p}=1-{\frac{d}{2r_{p}}}^{\;}$). Obviously, this process causes a mass loss. In a second step, the particle sizes are increased, in order to reproduce the volume fraction at its prescribed value. The growth coefficient relates the initial and the final particle radius to the final distance of the neighbouring particles ($\delta_{R}=\left(\frac{R_{p}-r_{p}}{R_{p}r_{p}}\right)d/2$). Combining these definitions, the final radius of the particles can be related to the initial radius, the overlapping coefficient, and the penetration coefficient, as follows:

(5) $$R_{p}=\frac{r_{p}}{1-\delta }\left(1-\delta_{p}\right),$$

The overlapping coefficient, *δ*, is the sum of the penetration ($\delta_{p}$) and the growth ($\delta_{R}$) coefficients: $\delta =\delta_{p}+\delta_{R}$. If δ=1, the aggregates are totally sintered (i.e., every aggregate merged into a single particle), whereas δ=0 indicates that particles are in point contact. The permissible values for the penetration ($\delta_{p}$) and growth ($\delta_{R}$) coefficients range from zero to δ.

**Figure 2:**
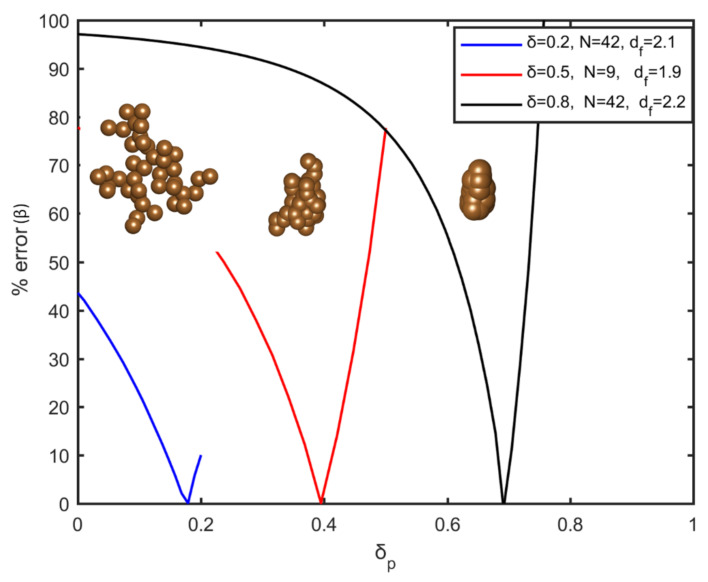
The percentage error in volume fraction $\left(\beta \right)$ varying with the penetration coefficient for different values of the fractal dimension, the number of particles in the aggregate, and the overlapping coefficient. A representative visualization of each aggregate structure is also shown.

The generation of sintered aggregates initiate with the determination of the volume fraction, the number of particles in the aggregate, the fractal dimension, and the overlapping coefficient. After the formation of each aggregate, a series of trial simulation scenarios are used to evaluate the values of $\delta_{p}$ and $R_{p}$. Evidently, each combination results in a different volume fraction ($f_{p,\delta }$). In order to determine the appropriate combination, for each aggregate the value of the penetration coefficient varies from zero to δ. For each $\delta_{p}$ value, the final radius (Equation (5)) is calculated. Finally, the volume fraction of the resulting system is compared with the initial volume fraction ($f_{p}$), ($\beta =100\cdot \left|f_{p,\delta }-f_{p}\right|/f_{p,\delta }$) and its dependence on $\delta_{p}$, as shown in Figure 2. The $\delta_{p}$ value with the smallest acceptable error is selected. Following the aforementioned technique, mass conservation is secured for each aggregate of the system, for the entire range of the overlapping coefficient. In Figure 2, the percentage error in volume fraction (β) is represented as a function of the penetration coefficient ($\delta_{p}$), for different values of the overlapping coefficient and the morphological characteristics of the initial aggregate. It is shown that a unique combination of $\delta_{p}$ and $R_{p}$ results in a system with the same volume fraction. This methodology, in addition to achieving the desired overlapping coefficient and volume fraction, has the advantage of being straightforward and fast during calculations. Needless to say, in real conditions the final structure during sintering may be different from that of the overlapping spheres, due to the appearance of neck effects and redistribution of mass that will eventually differentiate the structure from the one that is simulated here.

## 3. Effective Conductivity Calculation

The reconstructed aggregates that are obtained following the algorithm of the previous section are used as input to heat transport modelling. A constant temperature difference is imposed along the vertical axis of the nanofluid, whereas the rest of the boundaries are considered periodic. For the numerical solution of the heat transport equation, the Meshless Local Petrov–Galerkin (MLPG) method is used [41]. It has been shown to offer concrete advantages to more conventional methods in particulate systems with several contact areas, as is the case here. Differential equations are integrated into local subdomains, a fact that facilitates the increase of discretisation in regions of the geometries where steep gradients are developed. In the nanofluid case, the effective conductivity changes drastically at the interface of nanoparticles and the base fluid. The shape of the subdomains alters the performance of the method, with cubic sectors having been proved to increase the stability of the method [40]. The DC PSE approach is chosen as the trial function, while a step function is used as test function for the integration [42,43]. A set of cubic grids digitizes the domain and the integrals are calculated with the Gauss quadrature method [43]. In each $\Omega_{x}$ subdomain, the dimensionless weak form of the energy equation is given by the relation [43]:

(6) $$\left(k_{r}{}_{p}-1\right)\int_{\partial \Omega_{x}}^{\;}\Phi \nabla T_{\hat{n}}d\left(\partial \Omega_{x}\right)+\int_{\partial \Omega_{x}}^{\;}\nabla T_{\hat{n}}d\left(\partial \Omega_{x}\right)=0,$$

where Φ is a spatial step function defined as unit in the particle phase and zero elsewhere, and $k_{r}{}_{p}=\frac{k_{p}}{k_{f}}$ is the ratio of the conductivity of the particles to that of the base fluid. The solution of the heat transfer equation determines the temperature throughout the computational domain. Then, the calculation of the dimensionless effective conductivity is straightforward from $k_{eff}=\bar{\overset{\;}{\underset{S}{\int }}k\frac{\partial T}{\partial n}dS}$, where the surface S is vertical to the heat flow. A detailed description of the approach, the respective variables and integrals, the mesh construction, and the conductivity calculation can be found in a previous work by the authors [43].

This method is capable of calculating the effective conductivity of large particle systems. The aggregates are considered stationary and the heat conduction equation is solved within the computational domain. A typical simulation contains about 500–1000 particles organized into aggregates. Modelling of aggregates in heat transfer processes is performed with in-house meshless CFD methods implemented in Matlab kernels, as described in [38]. The computational time for the reconstruction of the aggregates is also provided in [38], along with comparison with other aggregation models. The effective conductivity calculations used herein have been shown in [43] to reduce the computational cost, compared with other numerical models and commercial software. A typical run for 1000 particles requires ~5 mil. nodes and ~1.5 ks on an Intel(R) Xeon(R) Silver 4116 CPU at 2.10 GHz using 12 cores.

Moreover, the present method can be extended to include calculations of the effective conductivity of different nanoparticle shapes. If the equation of the external surface of the particles is simple, the application is straightforward; otherwise the aggregation algorithm should be modified rather drastically, especially for non-convex surfaces.

The corresponding predictions of analytical models for the effective thermal conductivity are presented next. A well-known model for the conductivity of dispersed particles is Maxwell’s effective medium theory. Maxwell developed an expression for the effective conductivity, $k_{eff}$, of a suspension of solid spheres in liquid [51]:

(7) $$k_{eff}=\frac{k_{p}+2k_{f}+2\left(k_{p}-k_{f}\right)f_{p}}{k_{p}+2k_{f}-2\left(k_{p}-k_{f}\right)f_{p}}k_{f},$$

where $k_{f}$ is the conductivity of the base fluid, $f_{p}$ is the volume fraction of the particles, and $k_{p}$ is the conductivity of the particles. According to this equation, the process is apparently not sensitive to the size or the arrangement of the dispersed phase. However, this relation is not symmetric. By replacing $k_{p}$ with $k_{f}$ and $f_{p}$ with $\left(1-f_{p}\right)$, the effective conductivity calculation changes drastically [11]:

(8) $$k_{eff}=\frac{k_{f}+2k_{p}+2\left(k_{f}-k_{p}\right)\left(1-f_{p}\right)}{k_{f}+2k_{p}-2\left(k_{f}-k_{p}\right)\left(1-f_{p}\right)}k_{p},$$

Equation (7) accurately predicts well-dispersed particles at low volume fraction, while Equation (8) has been used to describe the conductivity of aggregate structures. In this perspective, the particles are considered as a solid network, with the base fluid enclosed in some regions. In any case, Equations (7) and (8) estimate the lower and upper bounds of the conductivity of an inhomogeneous medium, respectively [52,53].

The majority of the attempts to develop a model for the conductivity of colloidal clusters include a two-step approximation. The clusters are considered as spheres, with an effective conductivity ($k_{e}$) and an effective volume fraction ($f_{e}$). For the calculation of $k_{e}$, many relations from the effective medium theory have been applied [11,52,53,54]. The size of these effective particles is usually set equal to the radius of gyration of the aggregates [11,55]. In this case, the effective volume fraction ($f_{e}$), can be expressed as:

(9) $$f_{e}=\frac{4\pi }{3}\sum_{i}^{N_{c}}R_{g}{{}_{i}}^{3}{}^{\;},$$

where $N_{c}$ is the number of aggregates in the solution and ${R_{g}}_{i}$ is the (dimensionless) radius of gyration of each aggregate. The volume fraction of the solid phase inside the aggregates is $f_{i}=f_{p}/f_{e}$ [17]. In this work, $k_{e}$ is calculated from the upper limit (Equation (8)) by swapping $f_{p}$ with $f_{i}$, while the Maxwell relation is used (Equation (7)) in a second step, by replacing $k_{p}$ with $k_{e}$ and $f_{p}$ with $f_{e}$.

## 4. Results and Discussion

### 4.1. Comparison with Other Aggregation Models

**Figure 3:**
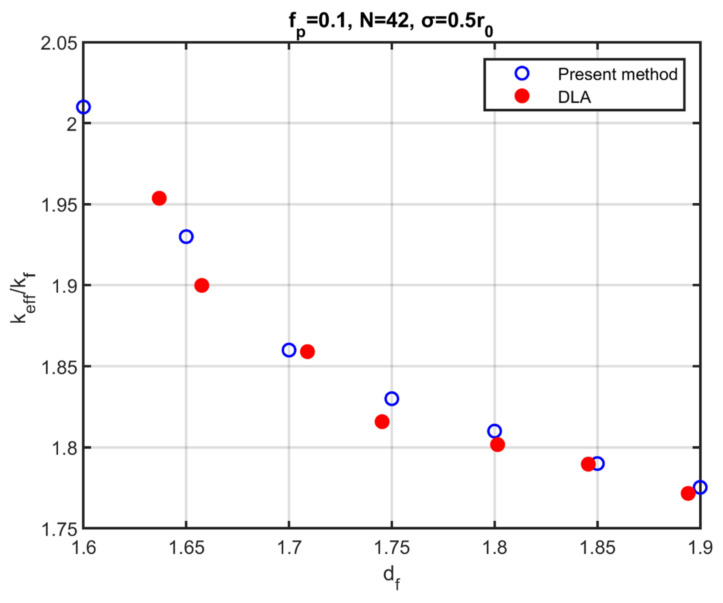
Effective thermal conductivity as a function of the fractal dimension of aggregates consisting of polydispersed particles by the present method (open, blue symbols) and by simulations from the DLA method (filled, red symbols).

Figure 3 shows the comparison between the effective conductivity of aggregates containing polydispersed particles, as extracted with the use of the method developed here, and the results of the DLA method. The volume fraction of particles is $f_{p}=0.1$ and the standard deviation of the particle size is $\sigma =0.5r_{0}$, where $r_{0}$ is the mean radius of the particles. The aggregates consist of Ν=42 particles and the thermal conductivity of the particles is considered $k_{r}{}_{p}=\frac{k_{p}}{k_{f}}$ larger than that that of the base fluid by a factor of 100. The simulation points are the averages of 10 realizations with the same morphological characteristics. In an earlier work by the authors, it was indicated that the mechanism of aggregation does not affect the effective conductivity for monodispersed particles. The same conclusion is drawn here for the case of polydispersed particles. However, the morphological characteristics of the aggregates appear to be significant for the effective thermal conductivity. Similar to the behaviour of aggregates of monodispersed particles, the thermal conductivity decreases with an increase in the fractal dimension.

### 4.2. Dependence of Conductivity on the Fractal Dimension, the Number of Particles in the Aggregate, and the Polydispersity of the Particles

In an earlier work by the authors [38], the effect of aggregation on the thermal conductivity of monodispersed particles was studied by varying the fractal dimension and the number of particles in the aggregate. A considerable increase in the thermal conductivity has been shown, even for aggregates consisting of a small number of particles. However, in the present work, a different behaviour is noticed upon the introduction of polydispersity in the particle size. The results are presented next.

**Figure 4:**
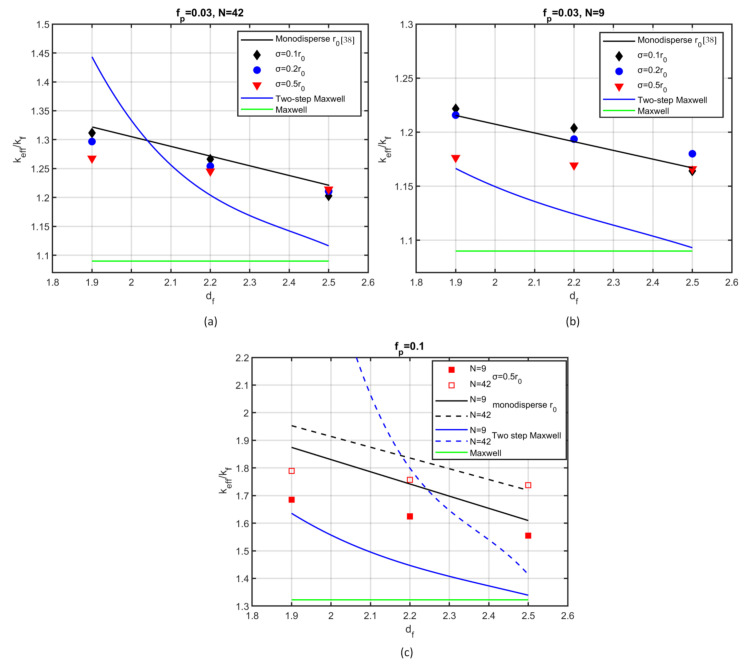
Effective thermal conductivity as a function of fractal dimension for: (**a**) volume fraction $\;f_{p}=0.03$ and number of particles per aggregate N=42; (**b**) $f_{p}=0.03$, N=9; (**c**) $f_{p}=0.1$, N=9 (solid lines, solid symbols), N=42 (dashed lines, open symbols). Black lines: monodispersed cases. Blue lines: two-step Maxwell model. Green line: Maxwell model. Symbols: polydispersed cases.

Figure 4 shows the dimensionless effective thermal conductivity of a nanofluid as a function of the fractal dimension, for two volume fractions, namely $f_{p}=0.03$ (Figure 4a,b) and for $f_{p}=0.1$ (Figure 4c). Two different values of the number of particles per aggregate are also investigated, namely N=42 (Figure 4a,c) and N=9 (Figure 4b,c). To enable a comparison, the mean radius of the particles for the polydispersed cases was set equal to the ones in corresponding cases of monodispersed particles. The standard deviation of the particle size varies from $\sigma =0.1r_{0}$ to $\sigma =0.5r_{0}$ in Figure 4a,b, whereas in Figure 4c it is kept at $\sigma =0.5r_{0}$. Figure 4 also shows the thermal conductivity predictions of two analytical models, namely, the two-step Maxwell (Equations (7) and (8)) and the single-step Maxwell model (Equation (7)). Every simulation point is the average of 10 realizations with the same parameters. The conductivity ratio of the nanoparticles and the base fluid is chosen to be 130 ($k_{r}{}_{p}=130$), which is a representative value for several practical nanofluids, such as water–Fe, engine oil–Al_2_O_3_, and water–CuO.

The effective conductivity decreased as the fractal dimension increased in all cases studied here. The level of reduction was affected by the polydispersity degree, the volume fraction, and the number of particles per aggregate. For cohesive aggregates, corresponding to relatively high fractal dimension ($d_{f}=2.5$), polydispersity did not affect the effective conductivity, whereas for smaller values of the fractal dimension and high polydispersity level, a notable variation was observed. More specifically, the thermal conductivity decreased with the increase of the deviation of the particle radius compared with monodisperse particle cases. However, a small deviation ($\sigma =0.1r_{0}$,$\;\sigma =0.2r_{0}$) affected the thermal conductivity only slightly (Figure 4a,b). A 10% reduction was observed for polydispersity degree $\sigma =0.5r_{0}$ and volume fraction $f_{p}=0.1$ (Figure 4c), whereas the reduction was only about 5% for volume fraction $f_{p}=0.03$ (Figure 4a,b).

The previous results are in contrast to the increase of the projected area of an aggregate as the polydispersity level is increased [32]. Particle size distribution affects heat transfer in two distinct ways. Large particles accelerate heat transfer, while small particles hinder it. According to the present results, the smaller particles act as regulators of heat transport; therefore, the effective conductivity is reduced. Experimental works have observed that polydispersity of the nanoparticles has a significant impact on the thermal properties of the nanofluid [56]. Specifically, the largest enhancement has been found for highly monodisperse particles [57].

The single-step Maxwell relation (Equation (7)) remains insensitive to the fractal dimension. However, the two-step Maxwell model, although affected by the fractal dimension and the number of particles in the aggregate, cannot predict the effective conductivity of such systems. It is worth noting that aggregation increased the effective conductivity of the nanofluid significantly in all cases studied. For highly polydispersed particles, when organized into aggregates consisting of, say, N=9 particles per aggregate, a 10% volume fraction (shown in Figure 4c) resulted in a 70% increase of the thermal conductivity. The higher the particles per aggregate, the higher the conductivity increase.

### 4.3. Effect of Sintering

**Figure 5:**
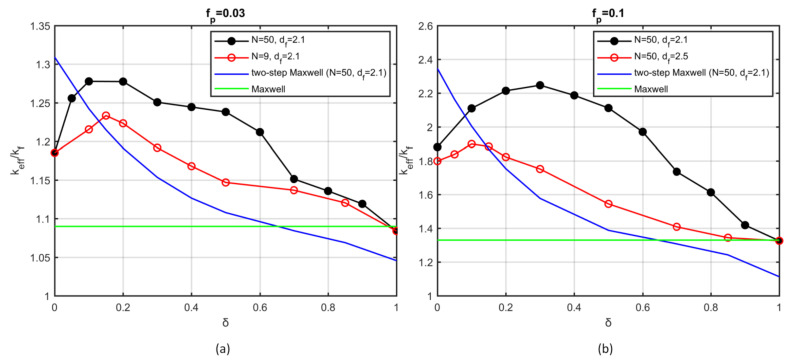
Effective thermal conductivity as a function of overlapping coefficient: (**a**) volume fraction $\;f_{p}=0.03;$ (**b**) volume fraction $\;f_{p}=0.1$. Solid, black circles: $N=50,\;d_{f}=2.1$. Open, red circles: (**a**) $N=9,\;d_{f}=2.1;$ (**b**) $N=50,d_{f}=2.5$. Blue lines: two-step Maxwell model. Green lines: Maxwell model.

Figure 5a,b portray the dependence of the thermal conductivity on the overlapping coefficient (*δ*) for nanoparticles that are organised into aggregates containing Ν=9 and Ν=50 particles, with volume fraction $f_{p}=0.03$ (Figure 5a) and $f_{p}=0.1$ (Figure 5b). The overlapping coefficient ranges from δ=0, which indicates particles at single-point contact, to δ=1, which corresponds to degeneration of the aggregate to a single particle. The corresponding thermal conductivity predictions of analytical models are also presented.

Upon the introduction of sintering, the effective conductivity is found to increase with the sintering level, up to a maximum value. Further sintering beyond that point has a negative effect on conduction until the value of Maxwell’s model is obtained, for δ=1. The value of the overlapping coefficient that offers the highest conductivity increase changes with the volume fraction and the number of particles per aggregate. A 15% maximum increase is shown for $f_{p}=0.1$, N=42, and δ=0.25 (Figure 5b). On the other hand, the conductivity calculated by the two-step Maxwell model decreases monotonically upon increase of the overlapping coefficient, due to the monotonic decrease of the radius of gyration ($R_{g}$).

The results showed that controlled aggregation and sintering can offer significantly improved thermal properties to nanofluids. From a physical point of view, higher values of the overlapping coefficient create an increased number of conduction pathways, which are also wider and longer, thus facilitating conduction along the macroscopic direction of heat transport. At the same time, the size of the aggregates decreases. For low sintering levels, the former factor prevails, whereas for high values of the overlapping coefficient, the aggregate tends to degenerate to an isolated body, thus reducing the thermal conductivity drastically.

## 5. Conclusions

The effect of particle size polydispersity and the sintering level on the thermal conductivity of aggregated nanoparticles was studied in the present paper. It was shown that both parameters examined here have the potential to change the heat performance of nanofluids drastically.

A method for reconstructing aggregates with the desired polydispersity degree was developed, satisfying simultaneously the requirements for certain morphological characteristics of the aggregate, namely, the fractal dimension and the number of particles in the aggregate. Particle sintering in aggregates was simulated for monodispersed cases and encoded as an overlapping mechanism in two steps: a penetration step and a growth step. In order to ensure mass conservation, the progression of each step was controlled through the minimisation of the error in the volume fraction of the sintered aggregate compared with the volume fraction of the initial aggregate. A meshless method with local refinement was used for the solution of the heat transfer equation and was found to be stable for the complex systems that were studied here. This is of key importance in the present problem as it allows using relatively large working domains that contain overlapping particles or particles at point contact with others and being able to extract statistically meaningful conclusions.

The effective thermal conductivity was calculated for aggregates that resulted from the present method of aggregation of polydispersed particles, then compared with the thermal conductivity of aggregates that were constructed with the Diffusion Limited Aggregation (DLA) method. The dependence of the effective thermal conductivity on the fractal dimension was found to be in good agreement with that in DLA method aggregates, which indicates that the proposed method produces aggregates that are thermally equivalent to those resulting from methods that describe the physical process of particle aggregation. Consequently, one can employ the present method for the investigation of the behaviour of nanofluids in heat transport problems, taking advantage of the increased simplicity of the aggregation algorithm and its rapid convergence to the final configuration.

The variation of the effective thermal conductivity was investigated over a wide range of fractal dimension values, number of particles per aggregate, and standard deviation of the particle size. Compared to fully dispersed particles, aggregation was shown to increase the thermal conductivity in all cases studied here. Small radius deviation does not substantially change the thermal conductance compared to monodispersed cases; however, a further increase of polydispersity leads to a clear reduction of the effective conductivity. On the contrary, strong polydispersity leads to an increase in the projected area, which implies an increase of heat transfer. A possible explanation for our result could be the existence of small particles within the aggregate that hinder heat transfer. This result is qualitatively confirmed by experimental measurements [56,57] according to which nanofluids consisting of particles with low polydispersity levels have higher heat performance compared to particles with high polydispersity.

The two-step Maxwell model predicts a monotonic decrease of the effective conductivity with increasing fractal dimension; however, large deviations from the numerical results were found for most of the cases examined here.

The effect of sintering of the aggregates was investigated and quantified as a function of the overlapping coefficient. Sintered aggregates have a lower effective size than the original aggregates, so a reduction in the effective conductivity should be expected. At the same time, sintering increases the heat conduction by forming larger heat pathways. This interplay yields a maximum in the thermal conductivity as a function of the degree of coalescence. The precise value of the overlapping coefficient that provides the highest conductivity increase depends on the morphological properties and the volume fraction of the initial aggregates. The present study indicates that the conditions of the production and dispersion of nanoparticles have a major impact on the thermal properties of the nanofluids. This is a possible explanation for the large deviations that have been observed between experimental works. Nanofluids with monodispersed particles, which are organized into aggregates with small overlapping, offer the highest heat transfer coefficient over the range of parameter values that were examined here. The results and conclusions of this work are also relevant to nanocomposite materials that contain polydispersed particle inclusions, which are organized in aggregates either at simple contact or in sintered form.

## Data Availability

Not applicable.
